# Roles of insolation forcing and CO_2_ forcing on Late Pleistocene seasonal sea surface temperatures

**DOI:** 10.1038/s41467-021-26051-y

**Published:** 2021-09-30

**Authors:** Kyung Eun Lee, Steven C. Clemens, Yoshimi Kubota, Axel Timmermann, Ann Holbourn, Sang-Wook Yeh, Si Woong Bae, Tae Wook Ko

**Affiliations:** 1 Division of Convergence on Marine Science, Korea Maritime and Ocean University, Busan, South Korea; 2 OST School, Korea Maritime and Ocean University, Busan, South Korea; 3 Earth, Environmental, and Planetary Sciences, Brown University, Providence, RI USA; 4 National Museum of Nature and Science, Tsukuba, Japan; 5 Center for Climate Physics Institute for Basic Science, Pusan National University, Busan, South Korea; 6 Institute of Geosciences, Christian-Albrechts-University, Kiel, Germany; 7 Department of Marine Science and Convergence Engineering, Hanyang University, ERICA, Ansan-si, South Korea

**Keywords:** Palaeoceanography, Palaeoclimate

## Abstract

Late Pleistocene changes in insolation, greenhouse gas concentrations, and ice sheets have different spatially and seasonally modulated climatic fingerprints. By exploring the seasonality of paleoclimate proxy data, we gain deeper insight into the drivers of climate changes. Here, we investigate changes in alkenone-based annual mean and *Globigerinoides ruber* Mg/Ca-based summer sea surface temperatures in the East China Sea and their linkages to climate forcing over the past 400,000 years. During interglacial-glacial cycles, there are phase differences between annual mean and seasonal (summer and winter) temperatures, which relate to seasonal insolation changes. These phase differences are most evident during interglacials. During glacial terminations, temperature changes were strongly affected by CO_2_. Early temperature minima, ~20,000 years before glacial terminations, except the last glacial period, coincide with the largest temperature differences between summer and winter, and with the timing of the lowest atmospheric CO_2_ concentration. These findings imply the need to consider proxy seasonality and seasonal climate variability to estimate climate sensitivity.

## Abstract (web summary)

How temperatures at different seasons differ in response to different forcings is not well known. Here, the authors reconstruct annual and seasonal sea surface temperatures in the East China Sea and show that they react differently to CO2 and insolation forcing on glacial-interglacial timescales.

## Introduction

Astronomical forcing is the primary external driver of glacial–interglacial cycles^1^. Climate/carbon and climate/ice-sheet feedbacks store and redistribute energy received from astronomical forcing, thus leading to the characteristic saw-tooth-like pattern of glacial–interglacial climate cycles^2^. Superimposed on orbital-scale variability is a continuum of climate variations that includes rapid changes during glacial inceptions and terminations^3^ as well as millennial-scale Dansgaard−Oeschger (DO) and Heinrich events^4,5^. The effect of these rapid climate shifts on regional and hemispheric sea surface temperatures (SSTs) has been extensively studied^1^.

Paleoclimate records are often compared with one another based on their phase relationships. Various processes contribute to the development of leads and lags between paleoclimate records, such as (i) the sensitivity to latitudinal variations in orbital forcing, (ii) the seasonality of regional climate feedbacks, (iii) regionally varying responses to different types of forcing, (iv) large-scale teleconnected climate patterns, and (v) the seasonality of proxy data. One or more of these processes may be involved, thereby complicating the interpretation of lead−lag relationships in terms of cause and effect. Prominent examples include the lead–lag relationship between Late Pleistocene atmospheric CO_2_ variations and reconstructed Antarctic^6^ or global mean temperature changes^7^. The reconstructed air temperature in Antarctica has been found to be nearly in-phase with boreal summer insolation but out-of-phase with local summer insolation; hence, it appears that the Antarctic climate is driven by boreal summer insolation. However, Antarctic temperature records derived from ice cores are biased towards austral winter^6^ and spring^8^. This is due to the seasonal cycle of snow accumulation, as well as the synchronicity of Antarctic temperature and local solar insolation. On millennial timescale, there have been many studies examining DO-type events^4^. Triggered in the North Atlantic region, these events had far-reaching atmospheric and oceanic impacts, affecting temperatures and hydroclimate in the Pacific Ocean^9–12^ outside the Atlantic realm. However, little is known about the seasonal relationships of Atlantic−Pacific climate millennial variations.

Disentangling the causes of Late Pleistocene climate variability, therefore, requires an in-depth understanding of the seasonal and spatial variations of forcing and climate responses. This effort is complicated by the fact that many paleo-proxies exhibit seasonal sensitivities^13^. Studies on the evolution of past SSTs based on different proxies revealed clear differences in the amplitude and timing of changes. For example, SST records from the western equatorial Pacific Ocean^14,15^, the eastern equatorial Pacific Ocean^16^, and Indian Ocean^17,18^ indicate that different proxies (foraminiferal Mg/Ca-based and alkenone-based SSTs) exhibit divergent trends for deglacial warming in tropical regions. One possible explanation is the different sensitivities of *Globigerinoides ruber* and alkenone producers to seasonal temperature variations^15–19^. Thus, knowledge of the seasonal sensitivities of SST proxies is crucial to elucidate climate processes and feedbacks on seasonal timescales.

In this work, we investigate the seasonal sensitivity of foraminiferal Mg/Ca and alkenone temperature records from the same core in the East China Sea located at the western margin of the subtropical North Pacific Ocean over the last four interglacial-glacial cycles. The Mg/Ca ratio- and alkenone-based SSTs reflect summer and annual mean temperatures, respectively. We further calculate winter temperature based on the reconstructed annual mean and summer temperatures. Comparisons of annual mean and seasonal (summer and winter) temperature variability with orbital forcing and greenhouse gas forcing indicate that changes in annual mean and seasonal temperatures differ in response to insolation and CO_2_ forcing.

## Results and discussion

### Proxy seasonality

We investigated high-resolution SST records at Site U1429 of the Integrated Ocean Drilling Program^20^ in the northern reaches of the Okinawa Trough (31°37.04′N, 128°59.85′E, water depth of ~732 m, Supplementary Fig. 1). The Kuroshio, a strong western boundary current of the subtropical North Pacific gyre, flows northward after entering the Okinawa Trough through the Yonaguni Depression between Taiwan and the Ryukyu islands, then exits the Okinawa Trough through the Tokara Strait to the south of the Kyushu (Supplementary Fig. 2). The core site is located to the north of the main axis of the Kuroshio Current and is affected by variations along one of its branches^21^ rather than being under the direct influence of the Kuroshio Current. This mid-latitude site with a high seasonality of SSTs (~11 °C) in comparison to low latitude locations has several advantages. The site is not subject to local upwelling, nor is it proximal to strong SST gradients associated with oceanic fronts. The site also has a relatively shallow water depth, which minimizes the effect of carbonate dissolution on foraminiferal shells and the corresponding Mg/Ca ratio. Moreover, this site records more than four interglacial–glacial cycles at ultra-high-resolution, thus providing detailed information of past warm and cold extremes in climate.

**Fig. 1:**
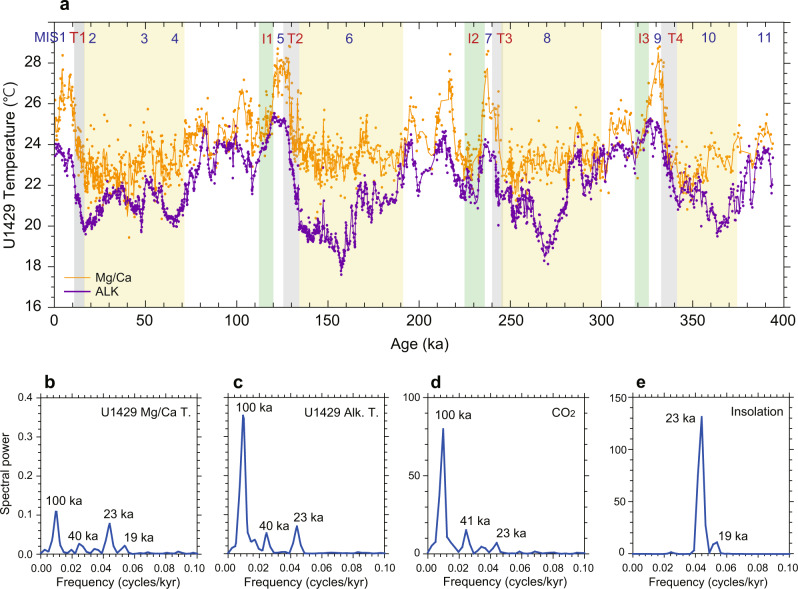
Reconstructed north-western Pacific sea surface temperature. **a** Alkenone-based temperature record for Site U1429 (three-point running mean, purple line) and *G. ruber* Mg/Ca temperature record for Site U1429 (three-point running mean, orange line) over the past 400 kyr. Power spectral density of **b** *G. ruber* Mg/Ca temperature, **c** alkenone temperature, **d** Antarctic composite CO_2_ concentration^49^ (Antarctic Ice Core Chronology 2012 age model), and **e** orbital insolation at the latitude of 31.6°N. Yellow, grey, and green shaded areas indicate the glacial period, termination, and inception, respectively. Glacial inception periods are denoted as I1, I2, and I3^3^, and glacial termination periods are indicated as T1, T2, T3, and T4^23^.

We compared high-resolution SST records of C_37_ alkenones (extracted from bulk sediment) and the Mg/Ca ratio of *G. ruber* (see “Methods”). Newly analyzed alkenone and Mg/Ca data for this study (see “Methods”) were added to the previously published temperature data^22^. The stable oxygen isotope ratios of benthic and planktonic foraminifera were also measured in the same samples^22^. The age model was constructed by aligning benthic foraminiferal δ^18^O values with the LR04 stack^23^, and further refined by linking the *G. ruber* δ^18^O values for Site U1429 with speleothem calcite δ^18^O records from Chinese caves^22,24^ (see “Methods”, Supplementary Fig. 3). Our records have an average resolution of approximately 100–200 years that resolve multi-centennial- to millennial-scale features over the past 400 kyr (Fig. 1). The extended U1429 SST records provide the opportunity to explore the effects of seasonality over the past four large-amplitude glacial-interglacial cycles in a unique region with a high SST seasonal contrast.

The alkenone-based and *G. ruber* Mg/Ca-derived SST records exhibit a pronounced and coherent variability on orbital timescales of ~23 kyr (precession), ~41 kyr (obliquity), and ~100 kyr (eccentricity) (Fig. 1a–c). The relative amplitude of the orbital-scale signals differs between the alkenone and Mg/Ca datasets, with alkenone SST presenting a substantially stronger ~100 kyr cycle (Fig. 1b, c) due to the considerably lower glacial-age temperatures compared to Mg/Ca SST (Fig. 1a). During interglacial periods (marine isotope stages (MISs) 5, 7, and 9), Mg/Ca-based SST varied by 3–4 °C at the precession band with almost the same amplitude as that of glacial–interglacial changes (Fig. 1a, b). In contrast, alkenone-based SST displayed relatively low amplitude variability (2–3 °C) during interglacial periods in comparison to glacial–interglacial changes (~5 °C) (Fig. 1a, c). In addition, the alkenone-based SST record exhibited very pronounced temperature minima at approximately 157, 268, and 364 kyr during the MISs 6, 8, and 10 glacial periods, well before the onset of glacial terminations (Fig. 1a). Interestingly, this feature was not captured in the *G. ruber* Mg/Ca-based SST record (Fig. 1a), which showed a much more stable glacial temperature range over MISs 2–4, 6, 8, and 10.

**Fig. 2:**
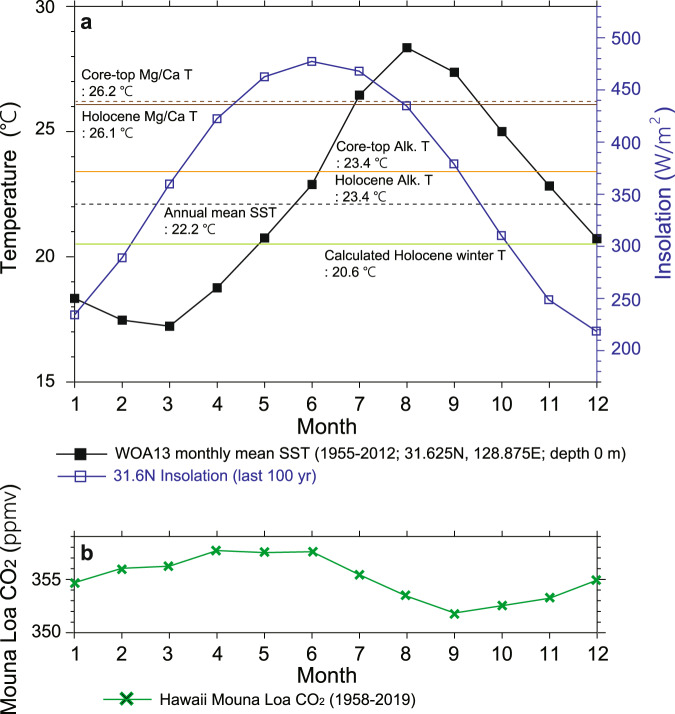
Modern monthly mean sea surface temperatures and insolation. **a** Monthly mean seawater temperature at the surface (black) (World Ocean Atlas 2013 (WOA 13); 0.25° resolution, observed from 1955 to 2012) and insolation calculated using the AnalySeries software^50^ (blue) at the latitude of site U1429 (31.6°N). **b** Monthly mean CO_2_ concentration at Mauna Loa (green) (NOAA/Earth System Research Laboratories, observed from 1958 to 2019).

**Fig. 3:**
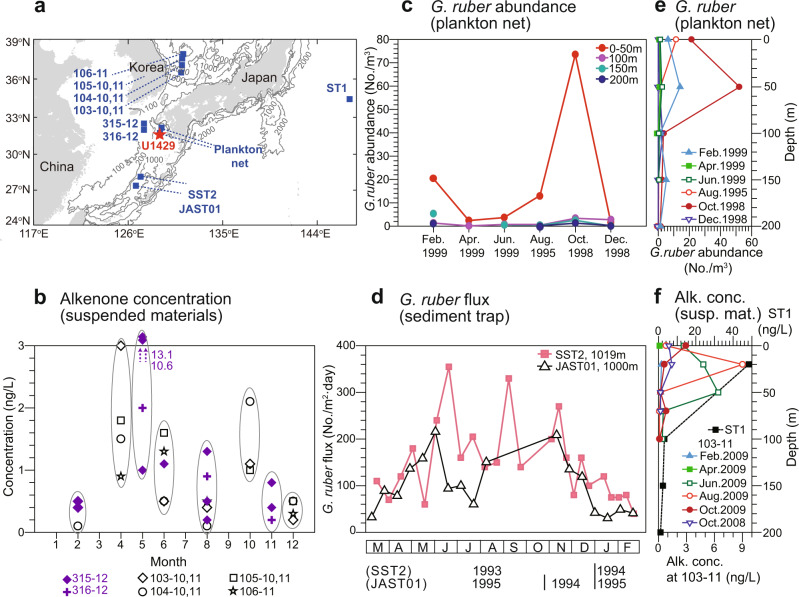
Seasonal and vertical variations of *Globigerinoides ruber* and alkenone producers. **a** Location of sampling sites in the East China Sea and nearby. **b** Variation in the alkenone concentration of suspended particles^32, 33^. **c** Variations in the abundance of *G. ruber* based on plankton net sampling in the East China Sea^28^. **d** Variations in the flux of *G. ruber* based on sediment trap records for the Okinawa Trough^26, 27^. Vertical distributions of the abundance of **e** *G. ruber*^28^ and **f** total C_37_ alkenone concentration in the northwest Pacific^32, 34^.

The reconstructed Mg/Ca temperatures were warmer than the alkenone temperatures for the entire period (Fig. 1a), thereby indicating a potential seasonal bias. At present, the seasonality is high (~11 °C) in this region with highest SST of 28 °C in August, and lowest SST of 17 °C in February and March (WOA 2013 SST dataset, Fig. 2a). The annual mean SST is 22 °C, which is close to the mean value of summer and winter. Previous studies based on suspended and sinking particles, core tops, and marine sediment cores from the East China Sea and nearby areas^25–30^ support the interpretation that the Mg/Ca ratios of *G. ruber* represent summer-biased (June−October) temperatures (Fig. 3a, c, d). Although there is a pronounced spring bloom of alkenone-producing coccolithophores^25,29,31–33^ in April and May (Fig. 3b), alkenone data in the region provide a reliable proxy for the annual mean temperature. Climatological SSTs in April and May (18−20 °C) are lower than the annual mean temperature (Fig. 2a). These seasonality interpretations are supported by comparing SST proxy values for the core top from Site U1429 and Holocene with the monthly mean SST at Site U1429 (WOA 2013) (Fig. 2a). Both coretop (26.2 °C) and Holocene (26.1 °C) Mg/Ca SST values are close to the mean value of SSTs for June to October (26.0 °C). By contrast, the coretop (23.4 °C) and Holocene (23.4 °C) alkenone temperatures are close to the annual mean SST (22.2 °C), although they are slightly higher than annual mean value, which is probably related to the relatively low production of alkenone in winter time (December−February) (Fig. 3b). The vertical distribution patterns of *G. ruber* abundance^28^ and the total C_37_ alkenone concentration^34^ in the upper water column reveal that these are high in the surface mixed-layer, thus indicating that *G. ruber* and alkenone-based records from marine sediments represent near-surface signals (Fig. 3e, f).

**Fig. 4:**
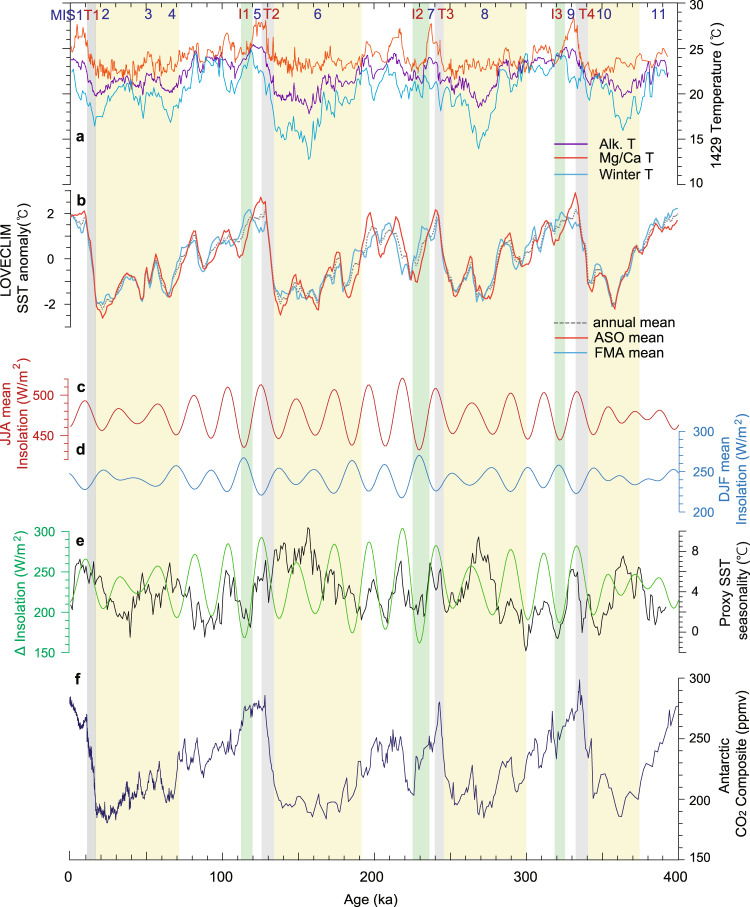
Time series of annual mean and seasonal sea surface temperatures (SST) and seasonality. **a** Comparison of *G. ruber* Mg/Ca-based summer temperature, alkenone-based annual mean temperature, and estimated winter temperature at Site U1429 over the past 400 kyr. **b** Seasonal temperatures from a transient LOVECLIM earth system model simulation^35^. **c** Summer (June−August) mean insolation at the latitude of Site U1429. **d** Winter (December−February) mean insolation at the latitude of Site U1429. **e** Difference in insolation between June−August and December−February and proxy SST seasonality (summer temperature minus winter temperature) variation. **f** Antarctic composite CO_2_ concentration^49^. Yellow, grey, and green shaded areas indicate the glacial period, termination, and inception, respectively. Glacial inception periods are denoted as I1, I2, and I3^3^, and glacial termination periods are indicated as T1, T2, T3, and T4^23^.

To further refine the seasonal interpretation of these records, we compared the proxy temperature reconstructions with the seasonal temperatures from a transient LOVECLIM earth system model simulation^35^ (see “Methods”) (Fig. 4a, b). Modelled SST records at the core location (31.5°N, 129°E) illustrate a phase difference between the monthly mean temperatures (Fig. 4b and Supplementary Fig. 4), which relate to monthly solar insolation changes in general (Fig. 4c, d and Supplementary Fig. 5). Changes in monthly mean solar insolation at 31.6°N (latitude for the core location) over the past 400 kyr shows that the insolation for June to August varied periodically with high amplitude. Furthermore, there is a 180° phase difference between summer (JJA) and winter (DJF) insolation. Since there is a lag of approximately two months between insolation and current SST data (Fig. 2a), we examined monthly mean modelled SSTs for summer (ASO) and winter (FMA). Comparison of the modelled SSTs with Mg/Ca SST shows that the Mg/Ca-based temperatures correlate well with the simulated ASO temperatures (Supplementary Fig. 6). Meanwhile, the alkenone data are highly correlated with simulated annual mean temperatures over the past 400 kyr (Supplementary Fig. 6). Comparison of alkenone SST with modelled SST for spring (April and May) over the past 400 kyr reveals a clear phase difference between them, which supports their interpretation as annual mean SST (Supplementary Fig. 4).

**Fig. 5:**
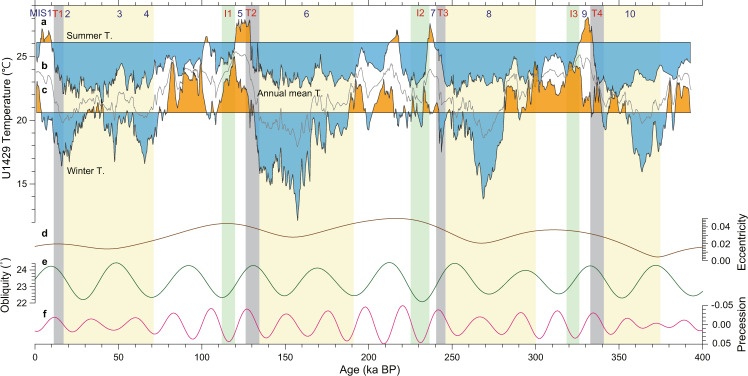
Time series of annual mean and seasonal sea surface temperatures (SST) and orbital parameters. **a** Mg/Ca-based summer SST (*T*_S_) at site U1429, **b** alkenone-based annual mean SST (*T*_A_), **c** calculated winter SST (T_W_ = 2T_A_ - T_S_), **d** eccentricity, **e** obliquity, and **f** precession. Three temperature time series are plotted on the same temperature axis. The horizontal line indicates the Holocene mean SST at Site U1429 for the summer and winter temperature time series. Orange and blue shaded areas indicate the values above and below the mean value, respectively, for summer and winter SSTs. Yellow, grey, and green shaded areas indicate the glacial period, termination, and inception, respectively. Glacial inception periods are denoted as I1, I2, and I3^3^, and glacial termination periods are indicated as T1, T2, T3, and T4^23^.

As the offset between the Mg/Ca and alkenone records is considered to be a good indicator of seasonality, winter temperatures (*T*_W_) were calculated (*T*_W_ ≅ 2*T*_A_ − *T*_S_) by assuming that the *G. ruber* Mg/Ca-based and alkenone-based SST records represent summer (*T*_S_) and annual mean (*T*_A_) SSTs, respectively. This calculation assumes that the annual mean SST is the mean of summer and winter temperatures. The seasonal behaviour of the proxy temperatures indicates that glacial winter temperatures were extremely cold, thus giving rise to a strong seasonality in SSTs during glacial periods (Fig. 5). The glacial–interglacial range for the estimated winter temperatures reached values of approximately 9 °C over the MIS 6–5 transition. Moreover, the winter temperatures remained relatively high during the entire interglacial periods (70−126, 190−240, 300−333 kyr, orange shading in Fig. 5), and were even higher than during the Holocene, whereas summer temperatures indicate a rapid cooling at approximately 120, 235, 330 kyr. The warm winter temperature during interglacials is consistent with previous climate model simulation results^36^.

### Roles of orbital forcing and CO_2_ forcing on seasonal SSTs

Our SST records indicate that the relative evolution of annual and seasonal SSTs through time depends on changes in insolation, greenhouse gases, and ice sheet volume. Here, seasonal differences in SSTs and associated forcings were examined for three different time intervals—interglacial, glacial termination, and glacial periods. The glacial inception periods denoted as I1 (112−120 ka), I2 (225−236 ka), and I3 (318-326 ka) mark the initiation of Northern Hemisphere ice sheet growth^3^, and glacial termination periods are indicated as T1 (11−17 ka), T2 (126−134 ka), T3 (240−246 ka) and T4 (333−341 ka)^23^. When precession minima and obliquity maxima are in phase, they strengthen summer insolation in the Northern Hemisphere (Fig. 5), and it could be expected that this would produce strong interglacial periods^36^. Conversely, when precession maxima and obliquity minima are in phase, they weaken summer insolation in the Northern Hemisphere, thus initiating glacial inceptions.

**Fig. 6:**
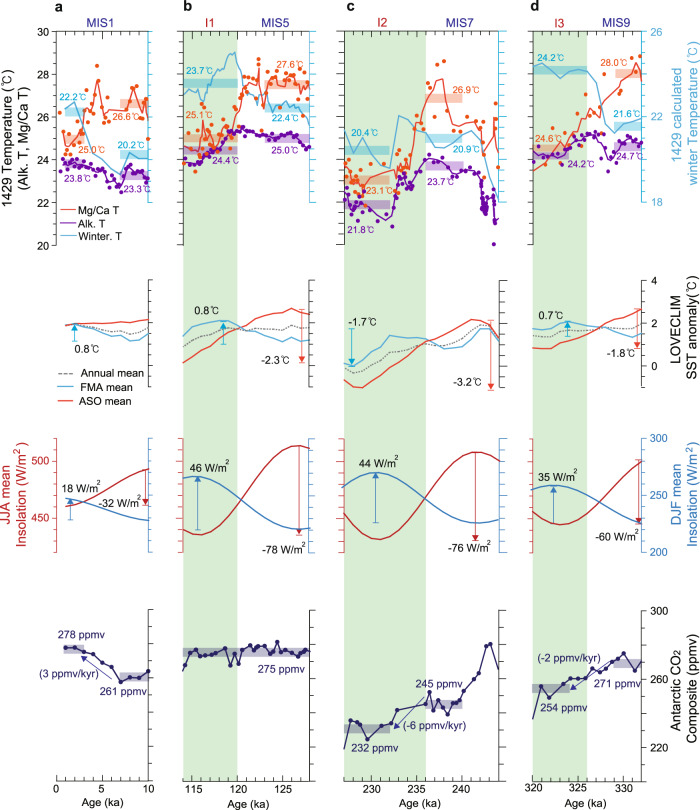
Reconstructed sea surface temperatures (SST) over the last four interglacials. Variations in proxy SSTs at Site U1429, LOVECLIM model SSTs, insolation for June to August and for December to February at the latitude of Site U1429 (31.6°N), and Antarctic composite CO_2_ concentration^49^ for the period of **a** 0−10 kyr, **b** 114−128 kyr, **c** 227−244 kyr, and **d** 320−332 kyr. Green shading indicates glacial inception period^3^.

Among the interglacials (Fig. 6), MIS 5 (115−128 kyr) is unique in that the CO_2_ concentration remained relatively constant (Fig. 6b). During this period, summer insolation (JJA) decreased by 78 W/m^2^, and winter insolation (DJF) increased by 46 W/m^2^. By responding to these seasonal insolation changes, model-simulated summer SSTs (ASO) decreased by 2.3 °C, and modelled winter SST (FMA) increased by ~1 °C. The proxy SST data for MIS 5 agree with the model-simulated changes. Mg/Ca-based summer SST decreased by approximately 3 °C, and alkenone-based annual mean SST decreased by ~1 °C. Meanwhile, reconstructed winter temperatures increased by ~1 °C. These results indicate that during MIS 5, when CO_2_ concentration was constant, the proxy and model seasonal temperatures correlate well with seasonal insolation changes in terms of both phase difference and the degree of changes. During other interglacial periods (MIS 1 and 9), the proxy and modelled seasonal SSTs exhibit similar relationships with climate forcing: when CO_2_ change was not significant, seasonal SST changes correspond to insolation changes (Fig. 6a, d). Meanwhile, the interglacial SST as a meanstate depend on the level of CO_2_ concentration. For instance, the reconstructed SSTs for MIS 7 (236−240 kyr) were lower (~1 °C) than for other interglacials (MISs 5 and 9) (Fig. 6c) which seems to be related to the lower CO_2_ concentration (by ~30 ppm) during MIS 7. At the beginning of glacial inceptions, summer-biased Mg/Ca-based SSTs at mid-latitudes of the Northern Hemisphere decreased rapidly, whereas alkenone-based annual mean SSTs decreased gradually and winter SST increased during glacial inceptions (Fig. 6). These comparisons support the interpretation that reduced summer insolation at high latitudes in the Northern Hemisphere led to a decline in summer temperatures during glacial inceptions (I1, I2, and I3).

**Fig. 7:**
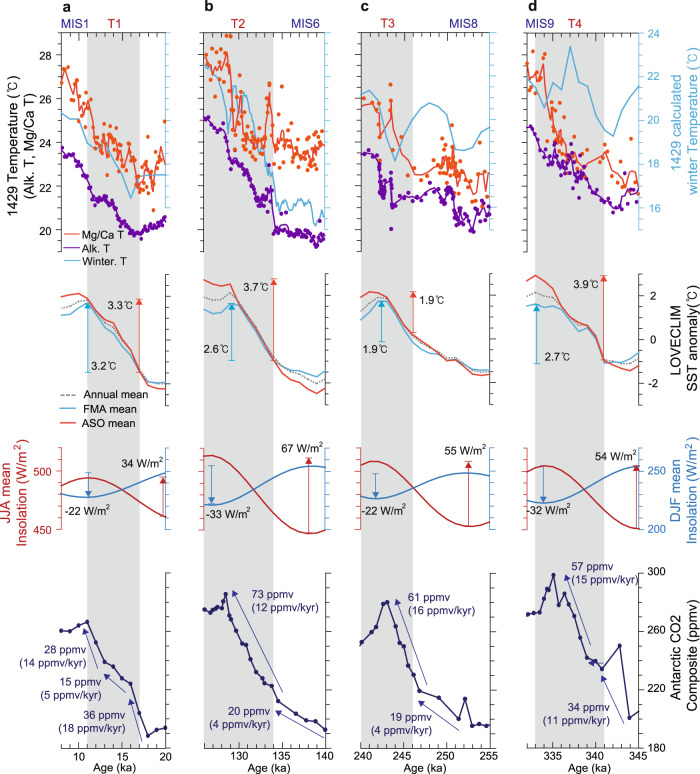
Reconstructed sea surface temperatures (SST) over the last four glacial terminations. Variations in proxy SSTs at Site U1429, LOVECLIM model SSTs, insolation for June to August and for December to February at the latitude of Site U1429 (31.6°N), and Antarctic composite CO_2_ concentration^49^ for the period of **a** 8−20 kyr, **b** 128−140 kyr, **c** 240−255 kyr, and **d** 332−345 kyr. Grey shading indicates a glacial termination period^23^.

Unlike interglacial periods, terminations are characterized by relatively small changes in insolation and by rapid and large changes in carbon dioxide concentration (Fig. 7). During Termination T2 (Fig. 7b), summer insolation increased by ~67 W/m^2^, and winter insolation decreased by ~33 W/m^2^. However, both modelled summer and winter SSTs increased by ~4 and ~3 °C, respectively. This is probably associated with the large increase in CO_2_ concentration by ~73 ppmv. Meanwhile, proxy-based summer SSTs did not change substantially (~1 °C) at the beginning of Termination T2, but subsequently increased rapidly (by ~3 °C) thereafter. The proxy-derived summer SST changes were consistent with summer insolation and CO_2_ changes. On the other hand, annual mean SST continued to increase (~5 °C) throughout the entire termination, which is consistent with CO_2_ changes, thereby supporting the view that changes in annual mean temperature are linked to CO_2_ changes. For Termination T3 (Fig. 7c), summer and annual mean SSTs increased stepwise at approximately 250 ka, and subsequently increased rapidly in tandem with CO_2_ changes from 240 to 246 ka. During Termination T1 (Fig. 7a), proxy-based SST, insolation, and CO_2_ changes differ from those of other termination periods (T2 and T3). The magnitude of seasonal insolation changes during T1 was relatively small compared to those of other terminations. The CO_2_ concentration increased substantially at the beginning of the termination (16−18 kyr), then increased gradually (13−16 kyr), and finally increased rapidly again (11−13 kyr). Increases in both summer and annual mean proxy temperatures show similar trends (Fig. 7a).

During the glacial periods, the simulated summer SSTs were dominantly influenced by the insolation cycles (Fig. 4b, c); this is particularly evident during MISs 3, 4, 6, and 8. However, Mg/Ca-based summer temperatures do not follow insolation cycles, unlike modelled summer SST (Fig. 4a–c). Comparison of changes in Mg/Ca SSTs and CO_2_ concentration shows that glacial summer temperature changes were not consistent with CO_2_ changes either (Fig. 4a, f). Meanwhile, alkenone-based annual mean temperature changes agree with CO_2_ changes on the orbital scale (Fig. 4a, f). In particular, when glacial greenhouse gases reach minima, annual mean temperatures also do the same. These results indicate that greenhouse gases play an important role in controlling the annual mean temperature during glacial periods. There is little phase difference between the two, indicating that annual mean temperature changes rapidly in relation to CO_2_ forcing. During glacial periods, the intensity of both summer and winter insolation decreased (Fig. 4c, d) and the seasonal difference in insolation also decreased (Fig. 4e). However, proxy-based SST records indicate that seasonality increased during glacial periods due to the cold winter temperatures (Fig. 4e).

To further document the relative roles of CO_2_ and insolation forcing on seasonal SSTs, correlation coefficients between the proxy-derived temperature, CO_2_ and insolation were calculated using the Ebisuzaki method^37–39^, which is suitable for serial correlation analysis (see “Methods”, Supplementary Table 1, and Supplementary Fig. 7). To perform this calculation, the records were divided into interglacial (MISs 1, 5, 7, and 9) and glacial (MISs 2–4, 6, 8, and 10) intervals, and correlation coefficients were calculated for the (i) total duration, (ii) interglacial periods, and (iii) glacial periods. The age definition of Lisiecki and Raymo^23^ was used to classify MISs, and transitional periods between glacial and interglacial intervals were not included in this calculation. A significant relationship between SSTs and insolation was only identified for interglacial summers, thereby supporting the notion that interglacial summer temperature is sensitive to insolation. A strong correlation (*r* = 0.83, statistically significant at the 95% level) was identified between CO_2_ and alkenone-based SSTs for the entire period, which suggests that SST sensitivity to CO_2_ was highest at the annual scale. Seasonal SSTs also showed relatively good correlations with CO_2_ over the entire period (*r* = 0.75 for summer and *r* = 0.67 for winter, statistically significant at the 95% level). However, glacial summer SST was not sensitive to CO_2_ (*r* = 0.14), while glacial winter temperature exhibited a good correlation with CO_2_ (*r* = 0.59, statistically significant at the 95% level). Hence, both insolation and CO_2_ forcing affected interglacial summer temperature, whereas glacial summer SST was not sensitive to either. Meanwhile, winter temperature was much more sensitive to CO_2_ forcing during glacial periods (*r* = 0.59) than during interglacial periods (*r* = 0.30).

**Fig. 8:**
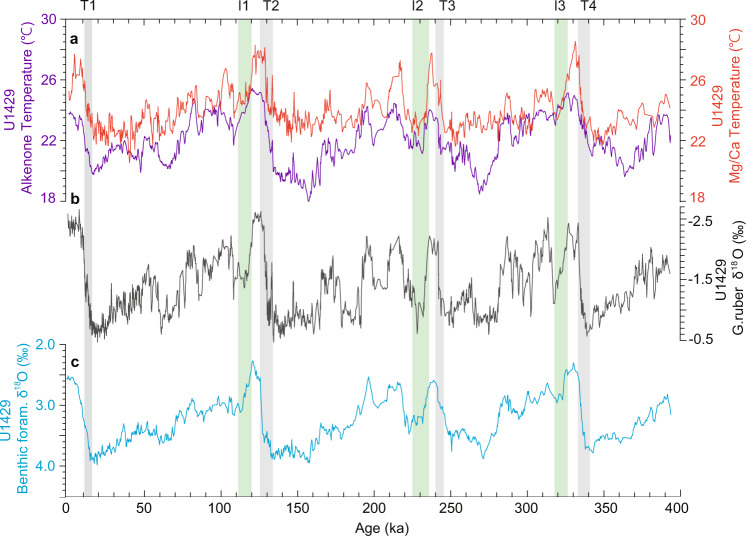
Time series of reconstructed sea surface temperatures (SST) and foraminiferal δ^18^O at Site U1429. **a** Alkenone-based temperature record (purple line) and *G. ruber* Mg/Ca temperature record (orange line). **b** *G. ruber* δ^18^O^22^, **c** benthic foraminiferal δ^18^O^22^. All records are three-point running mean. As all δ^18^O and SST proxies come from the same core material at U1429, there can be little stratigraphic uncertainty when inferring a tight correlation between different kinds of proxy records. Grey and green shaded areas indicate glacial termination and inception, respectively. Glacial inception periods are denoted as I1, I2, and I3^3^, and glacial termination periods are indicated as T1, T2, T3, and T4^23^.

In general, the growth and decay of large ice sheets over 100-kyr-cycles, as depicted in the deep-sea δ^18^O record, are characterized by long periods of ice growth/cooling followed by rapid ice decay/warming at terminations. Lisiecki and Raymo (2005) defined glacial terminations as the onset and end of rapid change in benthic foraminiferal δ^18^O. Compiled benthic foraminiferal δ^18^O records show that global ice volume reached maximum expansion just before glacial terminations^23^. To compare the timing of global ice volume changes with those of seasonal temperature changes, we examined the U1429 *G. ruber* δ^18^O and benthic foraminiferal δ^18^O records in comparison to seasonal SST variations. As all δ^18^O and seasonal SST proxies come from the same sediment succession, there can be little stratigraphic uncertainty, when inferring a tight correlation between proxy records. Our records indicate that ice decay did not occur substantially during the early stage of glacial terminations (T2 and T4 in Fig. 8), but rapidly intensified thereafter. This is consistent with proxy summer SST changes, but not the annual mean SST changes, which gradually increased throughout all of the glacial terminations, mainly controlled by CO_2_ changes. The alkenone-based annual mean SST at Site U1429 exhibited a pronounced temperature minimum approximately 20 kyr before the onset of glacial terminations during MISs 6, 8, and 10, whereas Mg/Ca-based summer SSTs did not. However, Termination T1 is exceptional. Alkenone-based annual mean SST did not exhibit clearly the early temperature minimum before the onset of Termination 1. Unlike the alkenone-based annual mean temperature minimum, a summer temperature minimum is not obvious during MISs 6, 8, and 10, although it occurred approximately 10 kyr before terminations T2, T3, and T4 (Fig. 8a). The timing of the early temperature minimum in the alkenone record was consistent with that of the lowest atmospheric CO_2_ concentration, thus indicating that CO_2_ forcing may be a more important factor than the direct and indirect influences of ice sheet-related forcing on annual mean SST changes during glacial periods.

### Seasonal relationship of Pacific–Atlantic millennial variations

The wavelet analysis of the SST data for Site U1429 reveals strong millennial-scale variations across the entire record (~400 kyr) (Supplementary Fig. 8). To isolate this scale of variability, a bandpass filter with limits of 300 years and 10,000 years was applied to all datasets. Our Mg/Ca-based and alkenone-based millennial-scale SST anomaly data (SSTA_mill_) showed a pervasive millennial variability in glacial temperatures (Supplementary Fig. 9). To investigate the teleconnections of seasonal SST variations on a millennial scale, we compared *G. ruber* Mg/Ca and alkenone SSTA_mill_ records to the Greenland ice-core temperature record, North Atlantic Pa/Th ratios, and atmospheric CO_2_ concentration changes (Supplementary Fig. 10). As other high-resolution records are only available for the last glacial period, these comparisons were only conducted for the last 70 kyr.

A comparison of the alkenone-based annual mean SST with the Greenland temperature record reveals that they co-varied in phase (*r* = 0.44, statistically significant at the 95% level; Supplementary Fig. 10, Supplementary Table 2). This implies that annual mean SSTs in the Northwest Pacific Ocean were linked to the DO activity captured in the Greenland record. Furthermore, we found a correlation between variations of the Atlantic Meridional Overturning Circulation (AMOC) in the North Atlantic Ocean^40^ and alkenone-based annual mean SSTs in the Northwest Pacific Ocean (*r* = −0.40, statistically significant at the 95% level; Supplementary Table 2). A weakened AMOC led to the simultaneous cooling of the North Atlantic and North Pacific (Supplementary Fig. 10), which agrees with paleoclimate model simulations^10,41^. However, the *G. ruber* Mg/Ca-based summer SST record at Site U1429 was not always consistent with the Greenland surface temperature record (*r* = 0.23) or AMOC record for the North Atlantic (*r* = −0.16). This could be partly due to a seasonal bias of the Mg/Ca-based summer season temperature record. Snow accumulation in Greenland is at its minimum during boreal summer, and the recorded Greenland surface temperature signals could be biased towards other seasons. Meanwhile, the weak correlation between Mg/Ca-based temperatures and North Atlantic Pa/Th ratios suggests that the linkage between summer temperature and AMOC changes is less significant. These indicate a seasonal relationship between Pacific–Atlantic climate millennial variations; the in-phase relationship between the Atlantic and Pacific DO variability appears to be stronger in the annual mean temperature record compared to that of the summer temperature record.

**Fig. 9:**
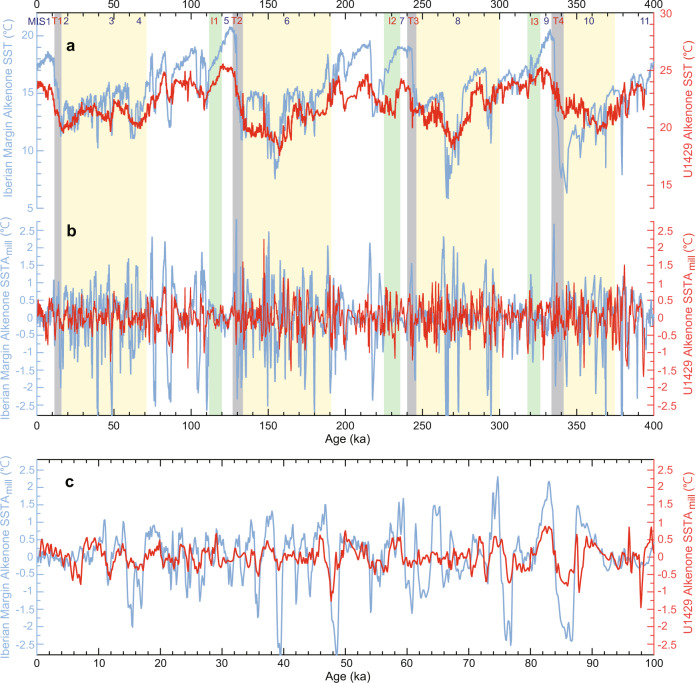
Comparison of alkenone-based sea surface temperature (SST) between the North Atlantic and North Pacific. **a** Comparison of orbital-scale and **b** millennial-scale variations in alkenone-based SST records from Site U1429 (red), and Iberian margin Cores MD01-2443 and MD01-2444 (blue)^42, 43^ over the past 400 kyr. Millennial-scale variability was extracted from all datasets by applying a bandpass filter with cuts of periods of 300 years and 10,000 years. Yellow, grey, and green shaded areas indicate the glacial period, termination, and inception periods, respectively. **c** Same as (**b**) but zoomed into the records over the last 100 kyr.

Interestingly, millennial variability in the alkenone record from the Northwest Pacific Ocean over the past 400 kyr exhibited a remarkable correlation with DO-scale variability in the alkenone-based SST record for the North Atlantic Ocean^42,43^ (Fig. 9). As δ^18^O variations for Site U1429 and the Iberian Margin are linked to speleothem chronology, these correlations were partly expected. However, it must be emphasized that SST data were not used in the development of the age model for Site U1429; only δ^18^O values of *G. ruber* were used. The alkenone-based SST record revealed that stadials in the North Atlantic were related to anomalously cold conditions in the Northwest Pacific (Fig. 9). This indicates a strong Atlantic–Pacific coupling over the past 400 kyr and the extension of DO-type SST variations in the North Pacific during MISs 6, 8, and 10. The in-phase relationship between Atlantic–Pacific millennial-scale annual mean SST variations supports the hypothesis that a strong and pervasive atmospheric bridge connected the Atlantic and North Pacific. This hypothesis also agrees with results from Atlantic freshwater perturbation simulations conducted using coupled general circulation models^10,41^. The standard deviation (1σ) of the alkenone-based SSTA_mill_ changes in the Atlantic on a millennial timescale was found to be ~1 °C^42,43^, whereas that in the Pacific reached 0.4 °C over ~400 kyr. The difference in the Atlantic/Pacific amplitude of the alkenone-based SSTA_mill_ variability along with the correlation between the reconstructed AMOC changes^40^ and the records from Site U1429 further support the notion of an Atlantic driver of Pacific DO-type millennial variability. A recent study^44^ suggested that on a multidecadal timescale, the warm SST anomaly in the Atlantic Ocean generates an atmospheric teleconnection to the North Pacific Ocean, which weakens the Aleutian low and subtropical North Pacific westerlies, and vice versa. The wind changes induce a subtropical North Pacific SST warming in a positive way. Our records support the coupling between Atlantic–Pacific linkages through atmospheric teleconnections on a millennial-scale.

Extended (400 kyr) and high-resolution (100−200 years) proxy records of *G. ruber* Mg/Ca-based (summer) and alkenone-based (annual mean) SSTs derived from the same sediment succession obtained from the north-western subtropical Pacific margin were investigated to address the following questions: (1) What are the seasonal responses of Pacific SST variations to climate forcing? (2) How did they differ during interglacial versus glacial time periods? (3) What is the seasonal relationship among Pacific–Atlantic climate millennial variations? Our results reveal that the timing and magnitude of maximum and minimum temperatures in interglacial−glacial cycles vary from season to season. Hence, the seasonality of SST proxy must be investigated first in order to understand the relationship between climate forcing and temperature. Changes in climate forcing within each interglacial−glacial cycle had their own unique characteristics over the past four cycles, and changes in annual mean and seasonal (summer and winter) temperatures also differ in response to forcing. This implies the need to consider seasonal bias, when evaluating climate sensitivity. Previous paleoclimate studies estimated equilibrium climate sensitivity^45^, i.e., the annual mean temperature change associated with CO_2_ radiative forcing, which is of crucial importance to climate change research. Recently, summer and winter anomalies associated with global greenhouse warming have been frequently encountered. To predict the potential impacts of future climate change, it is essential to understand changing seasonal anomalies. This paleoclimate study on the seasonal variation and response of SSTs to climate forcing at mid-latitudes will help to constrain future projections of climate changes.

## Methods

### SST reconstructions

Previous study presented the records of C_37_ alkenones and planktonic foraminiferal *Globigerinoides ruber* Mg/Ca ratios at Site U1429^22^. For this study, additional samples were analyzed: for alkenone SST reconstruction, 167 new samples were added to the previous 1735 samples^22^, and a total of 1902 samples was used in this study. For *G. ruber* Mg/Ca ratios, 358 new samples were added to the previous 1050 samples^22^, and a total of 1408 samples was analyzed. The new data added are mostly from intervals spanning glacial terminations and inceptions. For all proxy records, three-point running mean values were used. Our alkenone records, based on 1902 data points, had an average temporal resolution of approximately 100–200 years. For the Mg/Ca record, the average temporal resolution was 200–300 years (1408 data points).

The analytical methods for alkenones are the same as in ref. ^22^. Long-chain C_37_ alkenones were measured at the Korea Maritime and Ocean University. Bulk sediment samples (3 g) were taken from U1429 at 10 cm intervals for alkenone analysis. C_37_ alkenones were extracted from freeze-dried sediment samples. Organic compounds were extracted using an accelerated solvent extractor (ASE 200, Dionex) with a solvent mixture (CH_2_Cl_2_:CH_3_OH, 99:1 v/v) at high temperature (100 °C) and pressure (1500 psi). The extracts were cleaned by elution (3 × 500 μl CH_2_Cl_2_) through a silica cartridge. Saponification was performed at 70 °C for 2 h with 300 μl of 0.1 M KOH in CH_3_OH. The neutral fraction, containing the alkenones, was obtained by partitioning into hexane. After being concentrated under N_2_, the final extract was analyzed using a gas chromatograph (Agilent 7890 A) equipped with a flame ionization detector and a DB-1 column (60 m × 0.32 mm i.d.). Temperatures were calculated using the alkenone unsaturation index (U^K′^_37_) and the calibration equation of Prahl et al. (U^K′^_37_ = 0.034T + 0.039)^46^. The reproducibility of alkenone temperatures for replicate samples (*n* = 95) of a homogeneous marine sediment lab standard run during the project is better than ±0.1 °C at the 95% confidence level. Duplicate analyses from U1429 (*n* = 31) is ±0.4 °C at the 95% confidence interval.

The analytical methods for Mg/Ca ratio are the same as in ref. ^22^. Mg/Ca analysis of *G. ruber* sensu stricto (ss) was conducted for U1429 at intervals of approximately 10, 15, 25, or 30 cm, using splits of the same *G. ruber* size fraction used for measuring planktonic δ^18^O. Foraminiferal tests were cleaned following the reductive approach^30^. The metal/Ca ratios of samples were determined with a Thermo Scientific ELEMENT XR, a double-focusing sector field inductively coupled plasma mass spectrometer (ICP–MS) at the Mutsu Institute for Oceanography and determined with an ELEMENT 2 at the University of Toyama. The elements ^24^Mg and ^44^Ca were used to determine Mg/Ca ratio. The precision of the measurement was checked by replicate measurement (every five to six samples) of working standards. The relative standard deviation of the working standards was <2.6%. The accuracy of Mg/Ca ratios was confirmed by analysis of a reference standard, coral *Porites*, and the uncertainty is approximately ±0.05 mmol/mol (1σ). The value of 0.0131 mmol/mol was added for the Mg/Ca data measured at the University of Toyama for the difference of Mg/Ca between two laboratories. The contamination by diagenetic coating was monitored by measuring Mn/Ca ratios. Mn/Ca values of 99% of the samples were less than 0.5 mmol/mol. The average of the difference of Mg/Ca between 16 samples for the duplication test was 0.086 ± 0.149 mmol/mol, which was equivalent to 0.48 ± 0.36 °C. Because of the water depth of the core site (732 m) is above the modern lysocline (~1600 m) in the East China Sea, the preferential removal effect of Mg^2+^ from foraminiferal calcite on Mg/Ca values is likely negligible. The relationship between Mg/Ca and temperature of Mg/Ca = 0.38exp(0.09T)^47^ was used to calculate the temperature from *G. ruber* Mg/Ca.

### Transient LOVECLIM paleoclimate model simulation

To obtain a better understanding of the seasonality and timing of orbital-scale SST variations at Site U1429, we compared the reconstructed alkenone and Mg/Ca SSTs with annual mean and seasonal SST anomalies at a grid that covers the location of Site U1429 (31.5°N, 129°E), simulated by the LOVECLIM earth system model of intermediate complexity. It consists of a quasi-geostrophic three-layer atmosphere, a 20-level ocean general circulation model, a dynamic-thermodynamic sea-ice model, and a terrestrial vegetation model. LOVECLIM uses time-varying boundary conditions for orbital parameters, as well as CO_2_ and other greenhouse gas concentrations obtained from Antarctic ice cores, and an estimate of northern hemispheric ice-sheet orography and albedo changes^35^. The original transient model simulation covered the climatic evolution of the past 784 kyr, but our analysis here focusses only on the past 400 kyr.

### Age model

We used the age model constructed in a previous study^22^. An initial benthic age model was established by mapping the U1429 benthic δ^18^O to the global benthic stack^23^. The 100- and 41-kyr variances were then removed from planktonic *G. ruber* δ^18^O (benthic age model) by notch filtering, and then mapped to the composite cave δ^18^O record of Cheng et al.^24^. The key assumption of this age model was that millennial-scale variations in Chinese δ^18^O (a proxy for hydroclimate) are coherent with the measured δ^18^O of *G. ruber* in U1429. It should be noted here that we have not made any assumption on the timing of SST variations. Furthermore, this fine-tuning of the U1429 benthic age model is justified on the basis that they are highly coherent (>0.99 CI) with near-zero phase at the eccentricity, obliquity, and precession bands, which indicates that the U1429 benthic δ^18^O records in this age model are highly correlated with those based on benthic age models at an orbital timescale. Radiocarbon dates were measured at the depth of 22 cm and 35−37 cm^48^ at Site U1429 and calendar ages were 0.5 kyr BP and 1.37 kyr BP, respectively.

### Correlation coefficient and significance

We computed the correlation coefficient and significance between SST and other climate records by using the function “surrogateCor” implemented in the R-package ‘astrochron’^37–39^ with 10,000 Monte Carlo simulations. For orbital-scale comparisons, the data series were prepared prior to analysis by interpolation at 1,000 year intervals for the entire period of 0–393 kyr. Then, the period was divided into interglacial and glacial periods, and the correlation coefficient was calculated for each period (total, interglacial, and glacial periods). For millennial-scale comparisons, the data series were prepared by interpolation at 100 year intervals for the period of 14–71 kyr.

## Supplementary information

Supplementary Information

## Data Availability

Data relevant to this study are archived at the National Oceanic and Atmospheric Administration National Centres for Environmental Information (NCEI) (https://www.ncdc.noaa.gov/paleo/study/24810).
