# Supplementary material for: Roles of insolation forcing and CO2 forcing on Late Pleistocene seasonal sea surface temperatures
Source: Nat Commun. 2021 Sep 30;12:5742. doi: 10.1038/s41467-021-26051-y (PMC8484283; doi:10.1038/s41467-021-26051-y)
Supplement: Supplementary file 1 — Supplementary Information [file 41467_2021_26051_MOESM1_ESM.pdf]

Supplementary Information

Roles of insolation forcing and CO_2 forcing on Late Pleistocene seasonal sea surface temperatures

Kyung Eun Lee, Steven C. Clemens, Yoshimi Kubota, Axel Timmermann, Ann Holbourn, Sang-Wook Yeh, Si Woong Bae, Tae Wook Ko

Supplementary Fig. 1
Lee et al.

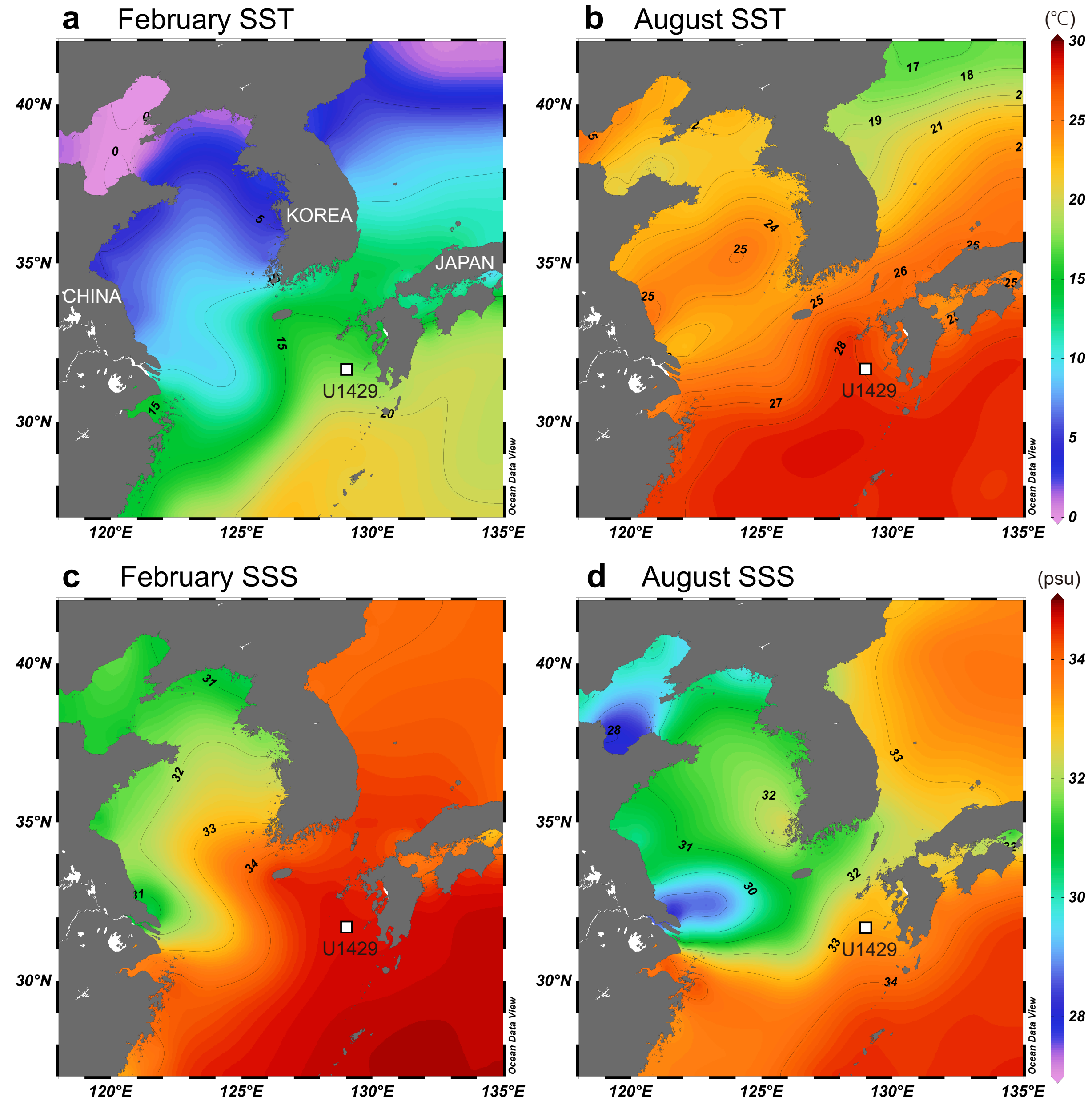

Supplementary Figure 1. Spatial distribution patterns of seawater temperature and salinity: Seawater temperature at a depth of 10 m during February and August **(a and b)**, and salinity **(c and d)** surrounding the study area. Colour shading represents monthly mean values of temperature and salinity for the period from 1955 to 2012 from the WOA (2013) dataset. White rectangle indicates the location of Site U1429.

Supplementary Fig. 2
Lee et al.

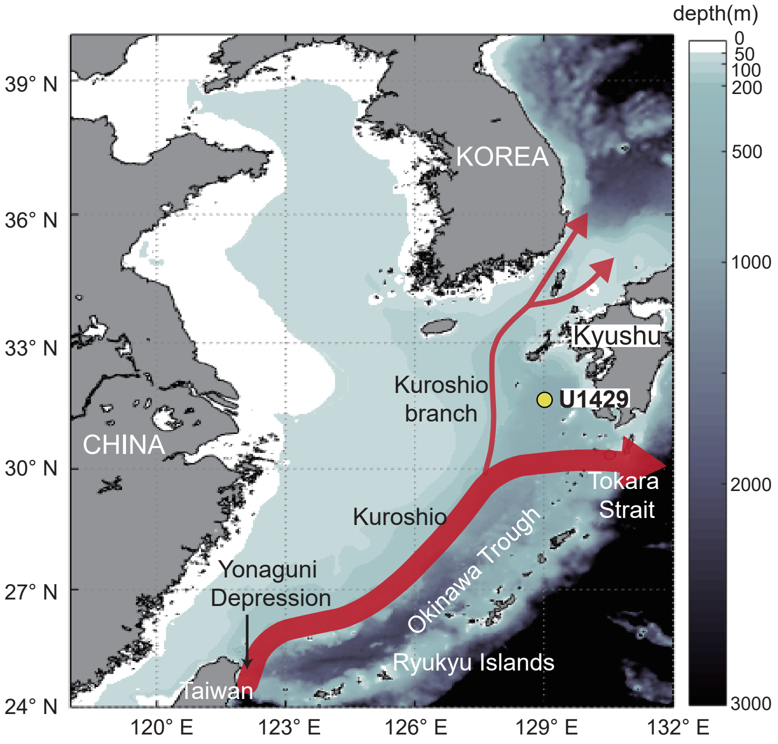

Supplementary Figure 2. Location map of Site U1429 with surface circulation patterns of the East China Sea.

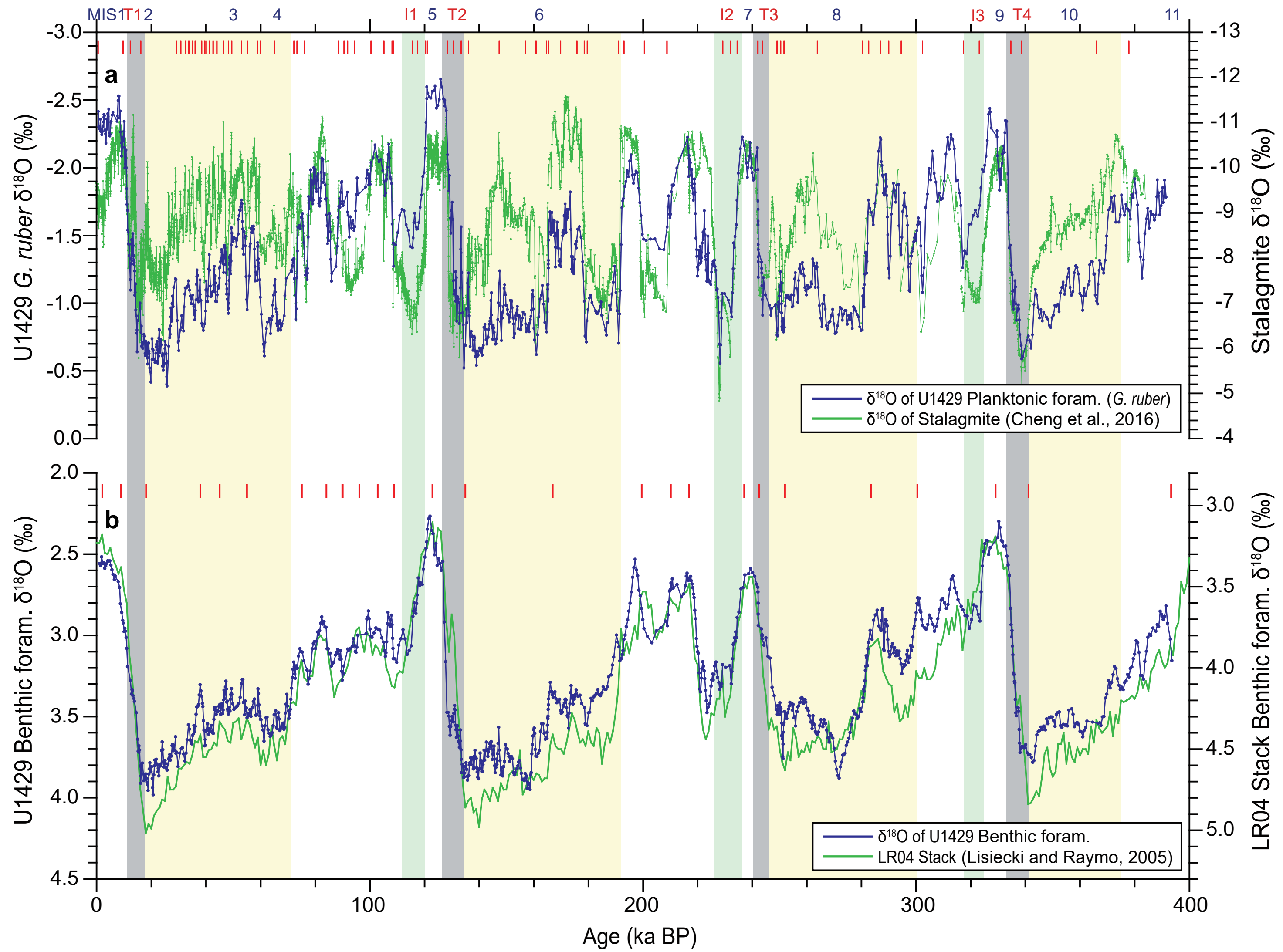

Supplementary Figure 3. Time series of foraminiferal δ^{18}O at Site U1429: (a) Comparison of *Globigerinoides ruber* δ^{18}O values at Site U1429 (blue) with stalagmite δ^{18}O values from Chinese caves (green)[1,2]. **(b)** Comparison of benthic δ^{18}O values at Site U1429 (blue) with stack benthic δ^{18}O values at LR04 (green)[2,3]. Red mark indicates the tie-point between G. ruber δ^{18}O values at Site U1429 and stalagmite δ^{18}O values for age control. Yellow, grey, and green shaded areas indicate glacial period, termination, and inception, respectively. Glacial inception periods are denoted as I1, I2, and I3[4], and glacial termination periods are indicated as T1, T2, T3 and T4[3].

Supplementary Fig. 4
Lee et al.

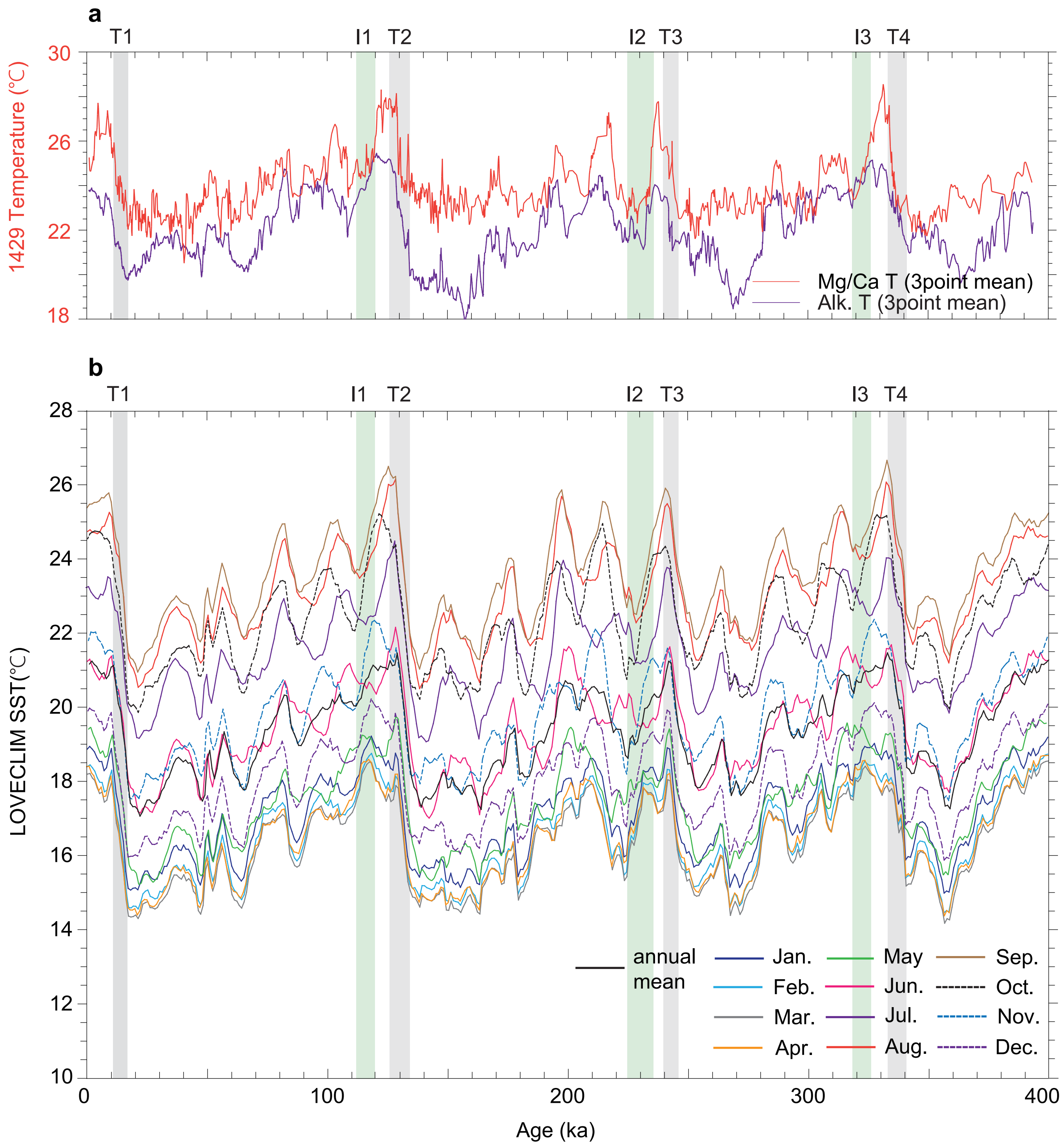

Supplementary Figure 4. Time series of reconstructed SSTs and simulated monthly mean SSTs: (a) Alkenone-based temperature record for Site U1429 (purple line) and *G. ruber* Mg/Ca temperature record for Site U1429 (orange line) over the past 400 kyr. **(b)** Annual mean and monthly mean SSTs from a transient LOVECLIM earth system model simulation[5] over the past 400 kyr. Glacial inception periods are denoted as I1, I2, and I3[4], and glacial termination periods are indicated as T1, T2, T3 and T4[3].

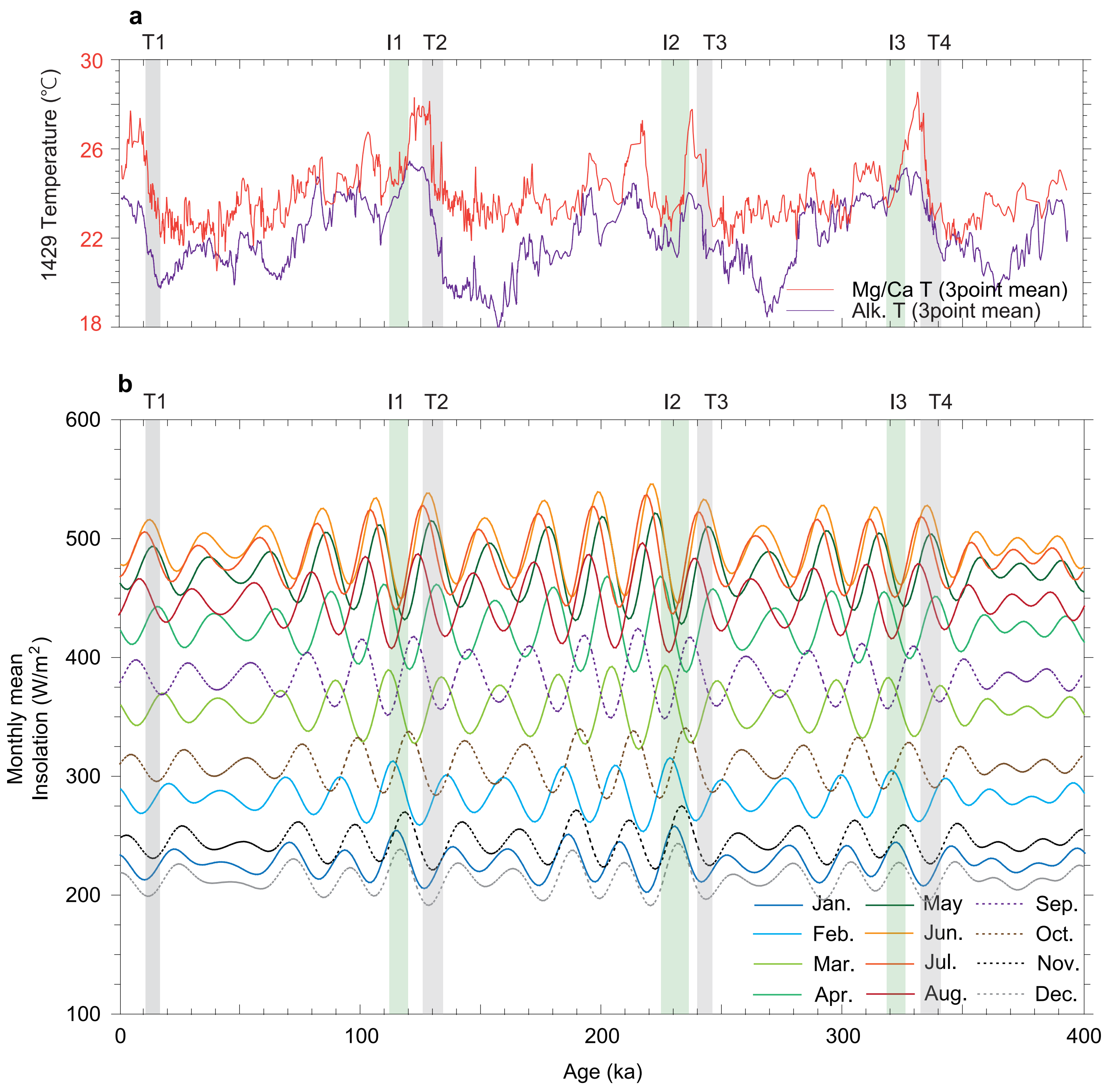

Supplementary Figure 5. Time series of reconstructed SSTs and monthly mean insolation: (a) Alkenone-based temperature record for Site U1429 (purple line) and *G. ruber* Mg/Ca temperature record for Site U1429 (orange line) over the past 400 kyr. **(b)** Monthly mean insolation over the past 400 kyr. Glacial inception periods are denoted as I1, I2, and I3[4], and glacial termination periods are indicated as T1, T2, T3 and T4[3].

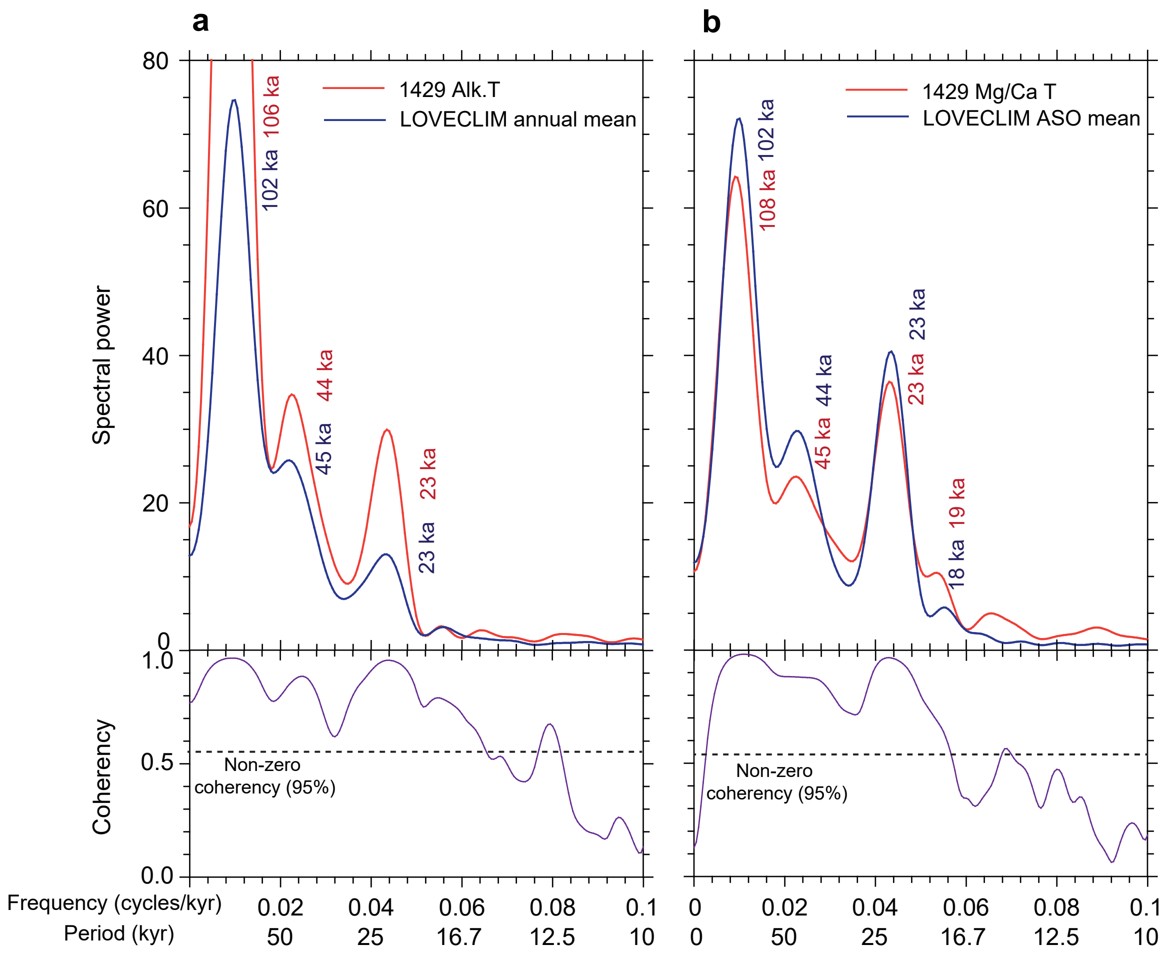

Supplementary Figure 6. Spectrum power and coherency between reconstructed SSTs and simulated SSTs: Coherency from cross-spectral analysis **(a)** between Site U1429 alkenone-based SST and LOVECLIM annual mean SST and **(b)** between Site U1429 Mg/Ca-based SST and LOVECLIM August to October mean SST.

Supplementary Fig. 7
Lee et al.

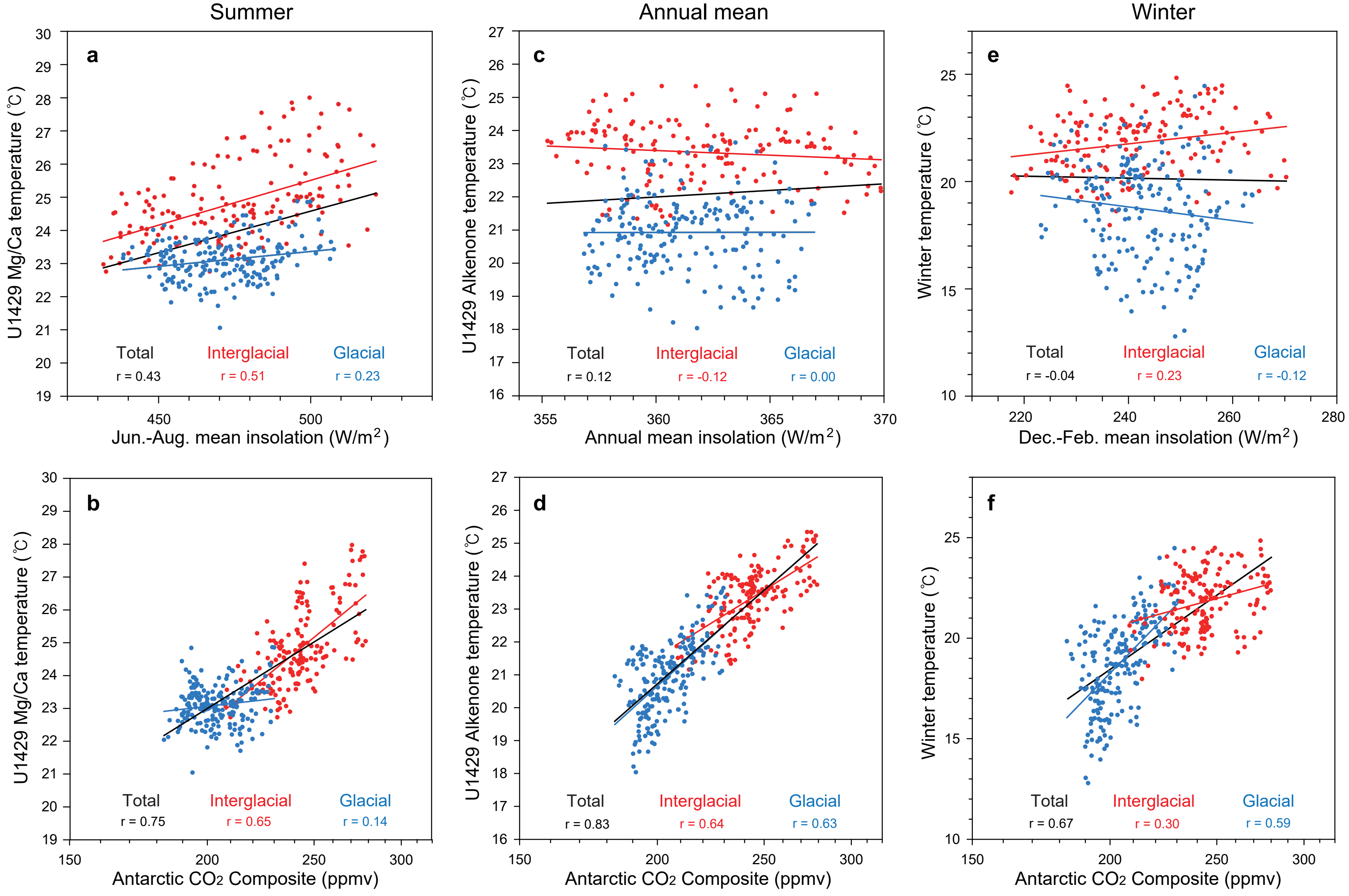

Supplementary Figure 7. Linear regression analysis between SST proxy records and forcing: (a) Mg/Ca-based SST at Site U1429 and mean insolation for June to August, and **(b)** CO_2[6]; **(c)** alkenone-based SST at Site U1429 and annual mean insolation and **(d)** CO_2; **(e)** calculated winter SST and mean insolation for December to February, and **(f)** CO_2. Each time series (three-point running mean) was interpolated at a 1-kyr interval before conducting the analysis. Red and blue dots represent interglacial and glacial periods, respectively.

Supplementary Fig. 8
Lee et al.

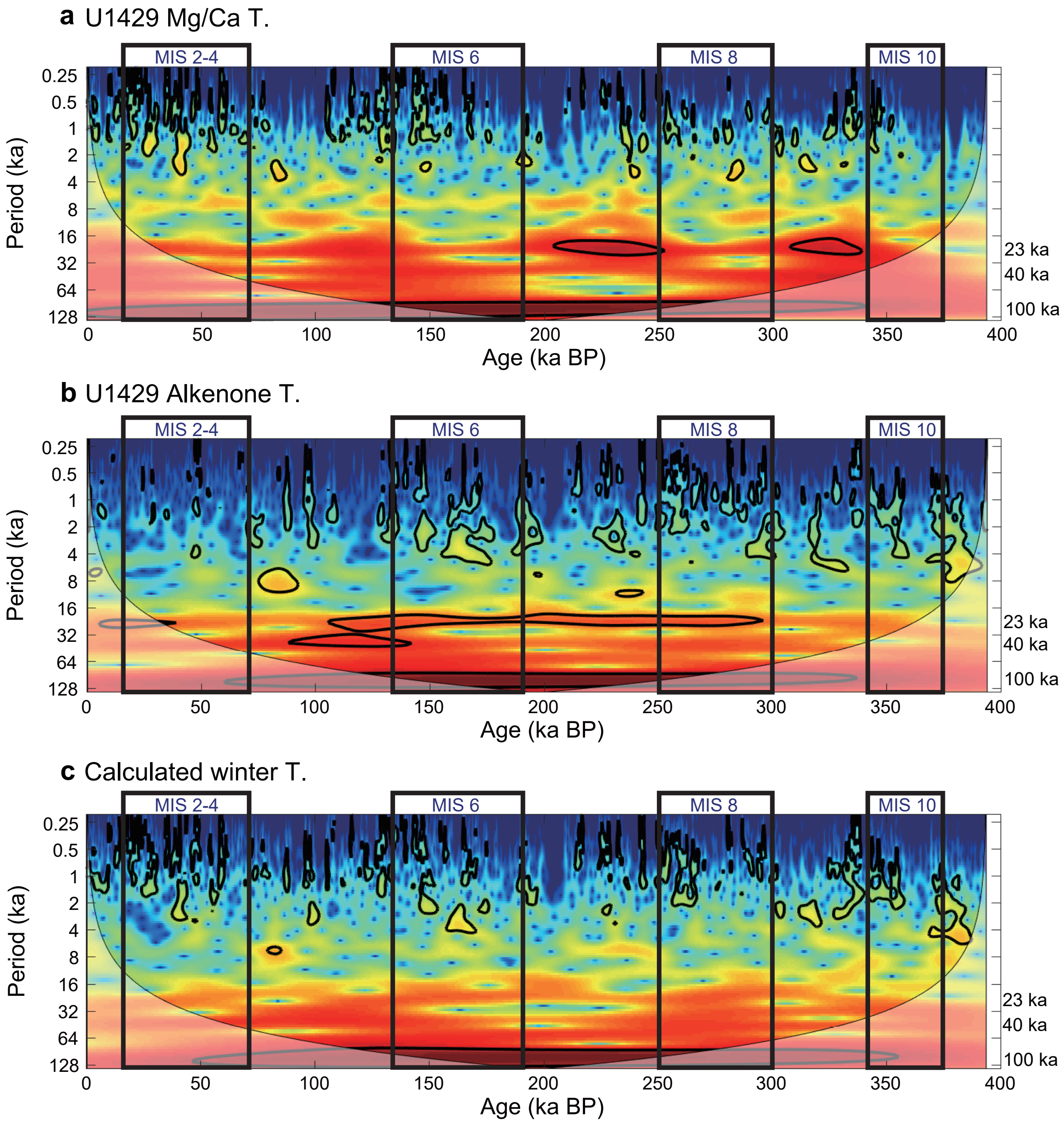

Supplementary Figure 8. Wavelet analysis: (a) Mg/Ca-based SST at Site U1429, **(b)** alkenone-based SST at Site U1429, and **(c)** calculated winter SST. Each time series was interpolated at a 0.1-kyr interval before conducting the analysis.

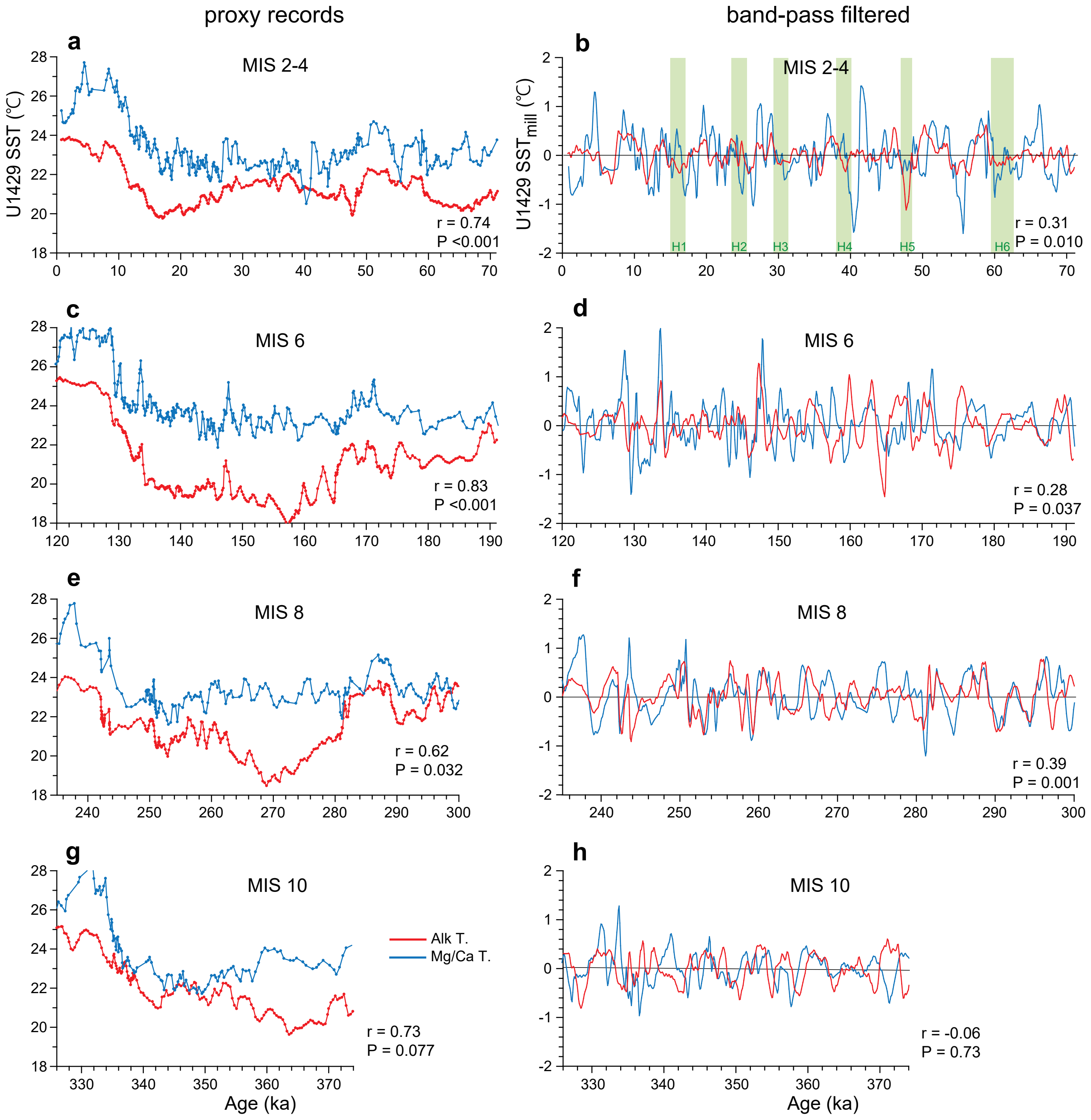

Supplementary Figure 9. Variations in SSTs over the last four glacial periods at Site U1429: (a, b) MIS 2–4 (0–71 kyr); **(c, d)** MIS 6 (120–191 kyr); **(e, f)** MIS 8 (235–300 kyr); **(g, h)** MIS 10 (326–374 kyr). (Left column) time series of three-point running mean alkenone-based (red) and Mg/Ca-based (blue) temperatures; (right column) millennial-scale variations in alkenone-based (red) and Mg/Ca-based (blue) temperatures. Millennial-scale variability was extracted by applying a bandpass filter with cuts of periods of 300 years and 10,000 years to time series of three-point running mean SST. Green shading indicates Heinrich events[7].

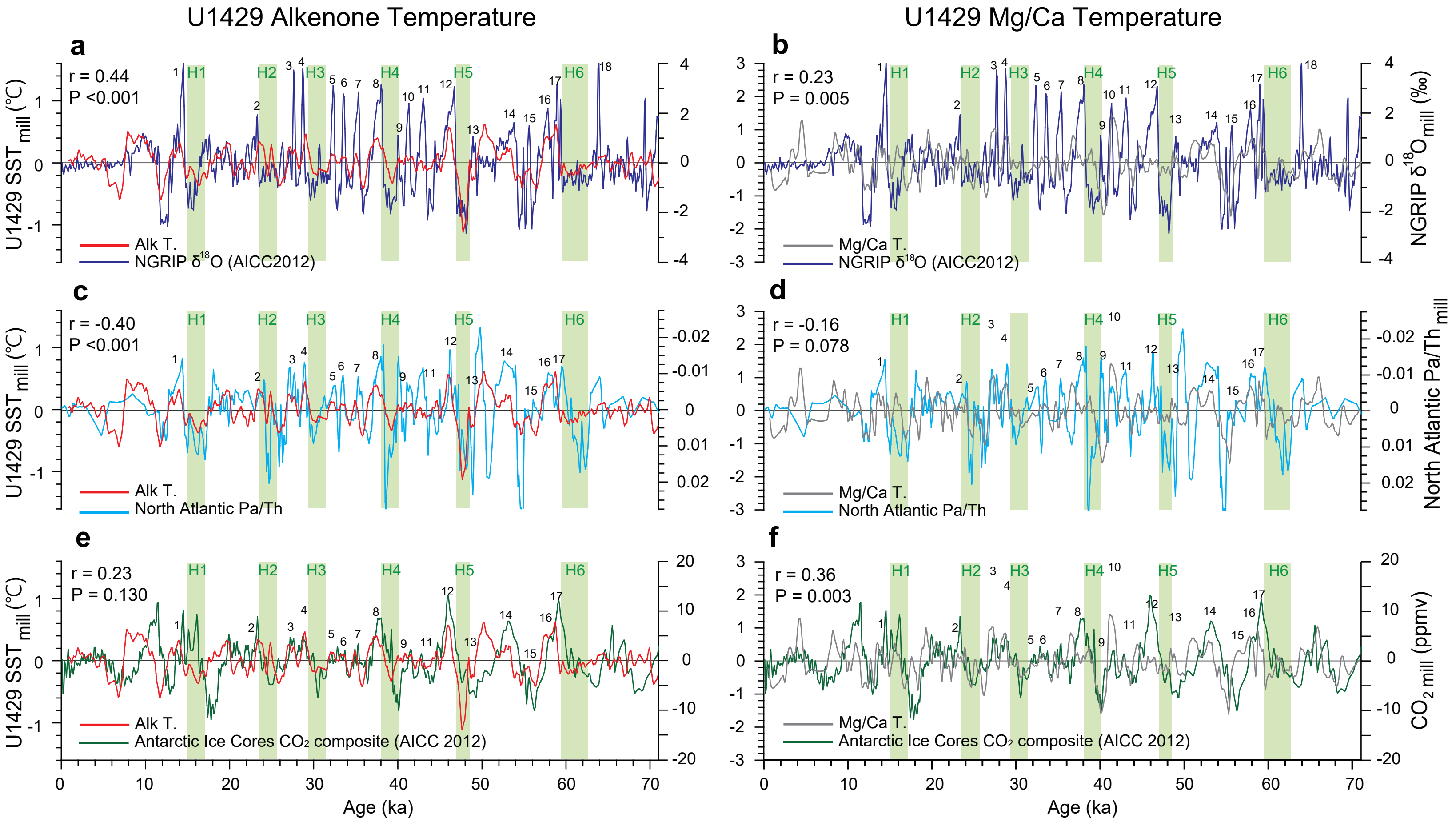

Supplementary Figure 10. Millennial-scale climate variations over the last glacial period: Comparison of millennial variations in alkenone-based SSTs (red, left column) and Mg/Ca-based SSTs (grey, right column) over the last glacial period (0–71 kyr) at Site U1429 with **(a, b)** the Greenland NGRIP $\delta^{18}O$ record (dark blue, AICC2012 age model)[8]; **(c, d)** North Atlantic Pa/Th isotope ratios representing changes of the AMOC (cyan)[9]; **(e, f)** Antarctic composite CO_2 concentration (green, AICC2012 age model)[6]. Millennial-scale variability was extracted from all datasets by applying a bandpass filter with cuts of periods of 300 years and 10,000 years. Green shading indicates Heinrich events[7].

Supplementary Tables

Supplementary Table 1. Correlation coefficient and statistical significance between orbital-scale variations in Site U1429 SST, insolation, and CO_2.

Forcing		Period	U1429 Mg/Ca T. (Summer)		U1429 Alkenone T. (Annual Mean)		U1429 Winter T.	
			r [a]	p [a]	r	p	r	p
Insolation	Jun.–Aug. mean Insolation[b]	Total[d]	0.43	0.015	0.21	0.387	0.04	0.847
		Interglacial[e]	0.51	0.019	0.12	0.692	-0.27	0.198
		Glacial[f]	0.23	0.078	0.15	0.397	0.08	0.607
	Annual mean Insolation	Total	0.29	0.349	0.12	0.667	0.00	0.999
		Interglacial	0.22	0.474	-0.12	0.734	-0.33	0.100
		Glacial	0.09	0.438	0.00	0.990	-0.02	0.878
	Dec.–Feb. mean Insolation	Total	-0.44	0.029	-0.21	0.308	-0.04	0.831
		Interglacial	-0.57	0.005	-0.20	0.495	0.23	0.308
		Glacial	-0.28	0.066	-0.20	0.225	-0.12	0.442
CO_2	log[CO_2][c]	Total	0.75	< 0.001	0.83	< 0.001	0.67	<0.001
		Interglacial	0.65	<0.001	0.64	<0.001	0.30	0.189
		Glacial	0.14	0.255	0.63	<0.001	0.59	0.005

[a] Correlation coefficient and statistical significance calculated by using the '*surrogateCor*' function of the *astrochron* R package[10-12].

[b] Insolation was calculated by using the Analyseries software[13] at 31.6 °N.

[c] Antarctic composite CO_2 concentration data from Bereiter *et al.*[6].

[d] Total: 0–393 kyr

[e] Interglacial: 0–10 ka, 72–125 ka, 192–239 ka, 301–332 ka, 375–393 ka

[f] Glacial: 18–71 ka, 135–191 ka, 247–300 ka, 342–374 ka

Supplementary Table 2. Correlation coefficient and statistical significance between millennial-scale variations over the last glaciation period.

	U1429 Mg/Ca T_{mill} (Summer)		U1429 Alkenone T_{mill} (Annual Mean)	
	r [a]	p [a]	r	P
NGRIP $\delta^{18}O_{mill}$ [b]	0.23	0.005	0.44	< 0.001
Pa/Th $_{mill}$ [c]	-0.16	0.080	-0.40	< 0.001
$CO_{2\ mill}$ [d]	0.36	0.003	0.23	0.130

[a] Correlation coefficient and statistical significance calculated by using the '*surrogateCor*' function of the *astrochron* R package[10-12].

[b] Data were from NGRIP Project Members[8].

[c] Data were from Henry et al.[9].

[d] Data were from Bereiter et al.[6].

Supplementary References

1. Cheng, H. et al. The Asian monsoon over the past 640,000 years and ice age terminations. *Nature* **534**, 640-646 (2016).
2. Clemens, S.C. *et al*. Precession-band variance missing from East Asian monsoon runoff. *Nature Communications* **9**, 3364 (2018).
3. Lisiecki, L.E., Raymo, M.E. A Pliocene-Pleistocene stack of 57 globally distributed benthic $\delta^{18}O$ records. *Paleoceanography* **20**, PA1003 (2005).
4. Ganopolski, A., Winkelmann, R., Schellnhuber, H.J. Critical insolation-CO_2 relation for diagnosing past and future glacial inception. *Nature* **529**, 200-203 (2016).
5. Timmermann, A., Friedrich, T. Late Pleistocene climate drivers of early human migration. *Nature* **538**, 92-95 (2016).
6. Bereiter, B. *et al*. Revision of the EPICA Dome C CO_2 record from 800 to 600 kyr before present. *Geophysical Research Letters* **42**, 542-549 (2015).
7. Hemming, S.R. Heinrich events: massive late Pleistocene detritus layers of the North Atlantic and their global climate imprint. *Reviews of Geophysics* **42**, RG1005 (2004).
8. NGRIP Project Members. High-resolution record of Northern Hemisphere climate extending into the last interglacial period. *Nature* **431**, 147–151 (2004).
9. Henry, L.G. *et al.* North Atlantic ocean circulation and abrupt climate change during the last glaciation. *Science* **353**, 470-474 (2016).
10. Ebisuzaki, W. A method to estimate the statistical significance of a correlation when the data are serially correlated. *Journal of Climate* **10**(9), 2147–2153. doi:10.1175/1520-0442(1997)010<2147:AMTETS>2.0.CO;2 (1997).
11. Meyers, S.R. Astrochron: an R package for astrochronology. http://cran.rproject.org/package=astrochron (2014)
12. Baddouh, M., Meyers, S.R., Carroll, A.R., Beard, B.L., Johnson, C.M. Lacustrine 87Sr/86Sr as a tracer to reconstruct Milankovitch forcing of the Eocene hydrologic cycle. *Earth and Planetary Science Letters* **448**, 62–68. doi:10.1016/j.epsl.2016.05.007 (2016).

13. Paillard, D., Labeyrie, L., Yiou, P. Macintosh program performs time-series analysis. Eos, Transactions American Geophysical Union **77**, 379 (1996).
